# Development and optimization of a high-throughput screening method utilizing *Ancylostoma ceylanicum* egg hatching to identify novel anthelmintics

**DOI:** 10.1371/journal.pone.0217019

**Published:** 2019-06-03

**Authors:** Laura Abriola, Denton Hoyer, Conor R. Caffrey, David L. Williams, Timothy P. Yoshino, Jon J. Vermeire

**Affiliations:** 1 Yale Center for Molecular Discovery, Yale University, New Haven, Connecticut, United States of America; 2 Skaggs School of Pharmacy and Pharmaceutical Sciences, University of California, San Diego, California, United States of America; 3 Department of Microbial Pathogens and Immunity, Rush University Medical Center, Chicago, Illinois, United States of America; 4 Cellular and Molecular Parasitology Training Program, Department of Pathobiological Sciences, University of Wisconsin, Madison, Wisconsin, United States of America; 5 Department of Pathology, University of California, San Francisco, California, United States of America; Universidade Guarulhos, BRAZIL

## Abstract

Hookworms remain a major health burden in the developing world, with hundreds of millions currently afflicted by these blood-feeding parasites. There exists a vital need for the discovery of novel drugs and identification of parasite drug targets for the development of chemotherapies. New drug development requires the identification of compounds that target molecules essential to parasite survival and preclinical testing in robust, standardized animal models of human disease. This process can prove costly and time consuming using conventional, low-throughput methods. We have developed a novel high-throughput screen (HTS) to identify anthelmintics for the treatment of soil-transmitted helminths. Our high-throughput, plate reader-based assay was used to rapidly assess compound toxicity to *Ancylostoma ceylanicum* L1 larva. Using this method, we screened 39,568 compounds from several small molecule screening libraries at 10 μM and identified 830 bioactive compounds that inhibit egg hatching of the human hookworm *A*. *ceylanicum* by >50%. Of these, 132 compounds inhibited hookworm egg hatching by >90% compared to controls. The nematicidal activities of 268 compounds were verified by retesting in the egg hatching assay and were also tested for toxicity against the human HeLa cell line at 10 μM. Fifty-nine compounds were verified to inhibit *A*. *ceylanicum* egg hatching by >80% and were <20% toxic to HeLa cells. Half-maximal inhibitory concentration (IC_50_) values were determined for the 59 hit compounds and ranged from 0.05–8.94 μM. This stringent advancement of compounds was designed to 1) systematically assess the nematicidal activity of novel compounds against the egg stage of *A*. *ceylanicum* hookworms in culture and 2) define their chemotherapeutic potential *in vivo* by evaluating their toxicity to human cells. Information gained from these experiments may directly contribute to the development of new drugs for the treatment of human hookworm disease.

## Introduction

Neglected tropical diseases (NTDs) are a group of 17 debilitating diseases that are strongly associated with poverty and major contributors to the global burden of infectious disease. Foremost among these are the five diseases caused by nematode worms, accounting for more than 80% of the global prevalence of NTDs and infecting more than 17% of the world’s population. Of the nematodes, hookworms remain a major health burden in the developing world with hundreds of millions currently afflicted by these blood-feeding parasites. The majority of human hookworm infections are caused by *Ancylostoma duodenale*, *A*. *ceylanicum*, and *Necator americanus* [1–3]. *Ancylostoma* infections are generally found in the temperate regions of the world, while *N*. *americanus* is more localized to tropical climates. For each hookworm species, the life cycle begins when eggs are deposited onto warm, moist soil via the feces of infected hosts. The eggs hatch, releasing first stage hookworm larvae (L1), which undergo successive molts to the infective third (L3) stage. Infectious L3 invade host skin and migrate to the lungs via the vasculature. After breaking out of the alveolar spaces and ascending the bronchial tree, the larvae are coughed up and swallowed by the host. Upon reaching the small intestine, the larvae molt to become adult worms and attach to the intestinal mucosa. There, the adult worms feed on host blood and tissue and begin to produce eggs. In heavily infected individuals with low dietary iron intake, the associated blood loss can rapidly lead to chronic hookworm disease characterized by severe anemia, malnutrition and growth/cognitive delay in children [1–6].

The standard treatment for intestinal nematodes, including hookworms, is chemotherapy with benzimidazole (BZ) anthelmintics (e.g. albendazole and mebendazole). These drugs which were developed in mid- to late 20th century, are clinically suboptimal, require multiple doses for maximum efficacy and are contra-indicated during early pregnancy. No safe, effective alternative therapies have yet been developed and approved for treatment purposes. In terms of both human and animal health, mass deworming programs have short-term benefits; rapid reinfection rates and declining efficacy of commonly used anthelmintics raise doubts about the long-term value of currently used chemotherapies as an effective means of disease control. A growing number of reports from laboratory and field studies around the world have documented decreased efficacies of the benzimidazole drugs currently in use against the human hookworms as well as soil-transmitted nematodes of agricultural and veterinary importance. Moreover, no single anthelmintic agent exists that is equally effective against all major soil-transmitted nematodes. Finally, efforts to develop safer, more effective anthelmintics approved for human use have not produced alternative therapies [7–9] Thus, there exists a need for the discovery of novel drugs and drug targets for the development of chemotherapies to treat intestinal worm infections caused by these 'resistant' populations of parasitic nematodes.

In the laboratory, *A*. *ceylanicum*-infected Golden Syrian hamsters (*Mesocricetus auratus*) are utilized as a model for human hookworm disease. Numerous groups have demonstrated that when infected with *A*. *ceylanicum*, hamsters exhibit disease pathologies, including delayed growth and anemia similar to the manifestation of disease in humans [10, 11]. Hookworm eggs appear in the feces and large numbers of viable eggs can be isolated. In this study, we have developed a high-throughput assay to detect hookworm egg hatching utilizing *A*. *ceylanicum* eggs obtained from infected Syrian hamsters. *A*. *ceylanicum* eggs are accurately distributed into the wells of microtiter plates containing compounds and incubated for 24 h. When nematode eggs hatch, chitinase is released into the media [12]. Chitinase is detected via cleavage of the fluorogenic chitinase substrate, 4-methylumbelliferyl-B-D-N,N',N"-triacetylchito-trioside (4-MeUmb), which produces a fluorescent product that is detected at 355/460 nm ex/em. The release of chitinase was determined to have a linear relationship with the number of eggs hatched/well. Compounds that are toxic to the *A*. *ceylanicum* eggs prevented their ability to hatch. Using this assay, we screened nearly 40,000 compounds and identified small molecule leads for further studies in drug development.

## Methods

### Production of *A*. *ceylanicum* eggs for HTS screening

Pooled fecal samples were harvested from Golden Syrian hamsters 14–21 days after infection with 75 infectious L3 stages of *A*. *ceylanicum* by oral gavage as previously described [10,13]. Hookworm eggs were purified from hamster feces using a density floatation method [14], and the mean number of eggs per milliliter was calculated by counting the mean number of eggs in 10 μL replicate aliquots by light microscopy prior to the distribution of eggs into microtiter plates with an automated Multidrop Combi Reagent Dispenser (Thermo Scientific).

### HTS (egg stages)

Small molecule compound libraries (NIH Clinical Collection, MicroSource Gen-Plus, MicroSource Natural Products, ChemBridge Small MW, ChemBridge DiverSet, Enzo kinase inhibitor, Yale Compounds, MicroSource Pharmakon 1600, ChemDiv Diversity, Rainforest Endophyte Extracts) were screened against *A*. *ceylanicum* eggs in our egg hatch assay. The libraries chosen represent focused compound collections with known bioactivity, natural products and extracts and libraries of unknown bioactivity that optimize diversity and chemical space, and retain drug like characteristics. The compound libraries utilized in this screen were formatted in 384-well plates as 10 mM stocks in dimethyl sulfoxide (DMSO) and have DMSO in the vehicle control wells. Except where noted, all reagents were purchased from Sigma. Sterile water (10 μL) was added to black-walled, clear bottom 384-well plates (Corning cat#3711) using the Multidrop Combi Reagent Dispenser. Compound (20 nL) was transferred from the compound library plate to the assay plate using an automated, multi-channel pipetting system (Aquarius, Tecan) with a 384-well pin tool (V&P Scientific). Freshly isolated hookworm eggs (75/well) were added in 10 μL volume of deionized water (ddH_2_O) using the MultiDrop Combi Reagent Dispenser. The eggs were kept in suspension during dispensing by mixing the reservoir contents with a pipette. Albendazole (ABZ; 1 μL of 200 μM diluted in water from a 10 mM stock in DMSO) was added to positive control wells. ABZ is the current gold standard for treatment of hookworm infections and a potent inhibitor of hookworm egg hatching *in vitro* (EC50 ≈0.5 μM). The final concentration of compound for primary screening was 10 μM and the final DMSO concentration was 0.1%. The assay plate was then incubated for 24 h at 27°C. After incubation, 4-MeUmb dissolved in DMSO at 1 mg/mLand diluted to 50 μM in water was added to a final concentration of 10 μM by the addition of 5 μL to each well by the MultiDrop. Screen plates were then centrifuged briefly at low speed and incubated for 1 h at 37°C. The reaction was terminated by the addition of 5 μL of 1M glycine/1N NaOH, pH 10.6, using the MultiDrop and the umbelliferone produced was measured in an EnVision plate reader (Perkin Elmer) (355/450nm ex/em).

Hatched eggs will release chitinase into the well and have a high fluorescence signal relative to unhatched eggs. Data was processed using IDBS ActivityBase screening data management software. Inhibition of hookworm egg hatching was calculated with the formula: 100 –(((sample–positive control mean) / (negative control mean–positive control mean))* 100). The “effect” of compound treatment measured is the inhibition of hookworm egg hatching. We observed ~90% viability of control *A*. *ceylanicum* eggs/larvae in the presence of ≤0.1% DMSO (negative control). Hit compounds were re-arrayed and confirmed at 10 μM. Active compounds were visually confirmed by microscopic evaluation to rule out compounds acting as chitinase enzyme inhibitors. Select compounds from the hit pick were run in a 6-point dose-response series at final assay concentrations of 10–0.04 μM in duplicate on two different days to determine the half-maximal inhibitory concentration (IC_50_) values. Normalized data were fitted to a dose response curve utilizing GraphPad Prism software with a nonlinear regression, 4 parameter logistic model.

### HeLa cell toxicity assay

Cells (20 μL) were plated into sterile black-walled, clear bottom, tissue culture treated, 384-well plates (Corning cat#3712) using the MultiDrop at a density of 400 cells/well. Plates were then centrifuged at 46 *g* for 10 sec and incubated overnight at 37°C in a humidified 5% CO_2_ incubator. Compound (20 nL) was transferred from the compound source plate to the cell assay plate using an Aquarius (Tecan) with a 384-well pin tool (V&P Scientific). The final concentration of compound and DMSO was 10 μM and 0.1%, respectively. Puromycin (2.5 μg/mL final added in 1 μL of solvent) was added as a positive control. Assay plates were centrifuged at 46 *g* for 10 sec and incubated for 72 h at 37°C in a humidified 5% CO_2_ incubator. CellTiter-Glo (Promega) was used to measure cell viability in the assay wells according to the manufacturer’s instructions. CellTiter-Glo reagent (20 μL/well) was added to the assay plates using the MultiDrop dispenser. The plates were shaken on a Thermomixer R (Eppendorf) at 1,100 rpm for 1 min and incubated in the dark for 10 min at room temperature. Luminescence was measured in the Envision plate reader with 0.3 second sampling time per well. Wells displaying cytotoxicity have lower luminescence signals relative to the vehicle control wells. Raw data (luminescence counts per second) were normalized to Percent Effect by the formula 100 –(((sample–positive control mean) / (negative control mean–positive control mean))* 100). The toxicity activity threshold was established as the median of the negative control wells + 3 standard deviations.

### Ethics statement

In his research lab at Yale Dr. Vermeire has a room dedicated to handling BSL 2 parasites, including hookworm. Dr. Vermeire has applied for and received IACUC-approved use of the *Ancylostoma ceylanicum* life cycle with the Yale IACUC board. Parasites at various states of maturity can be harvested from these hosts. Yale’s Institutional Animal Care and Use Committee annually review the Center’s vertebrate animal protocols. The Association for Assessment and Accreditation of Laboratory Animal Care (AAALAC) accredits Yale annually.

Yale’s Assurance Number: D16-00416

Approval Period: May 15, 2015-May 31, 2019

Yale animal researchers may review the Assurance by contacting the Office of Animal Research Support (OARS) at 203-785-5992.

Yale’s USDA License and Registration Number: 16-R-0001

Registration Period: March 20, 2017-March 20, 2020

AAALAC Accreditation: March 15, 2016

## Results

### Assay optimization

**Fig 1:**
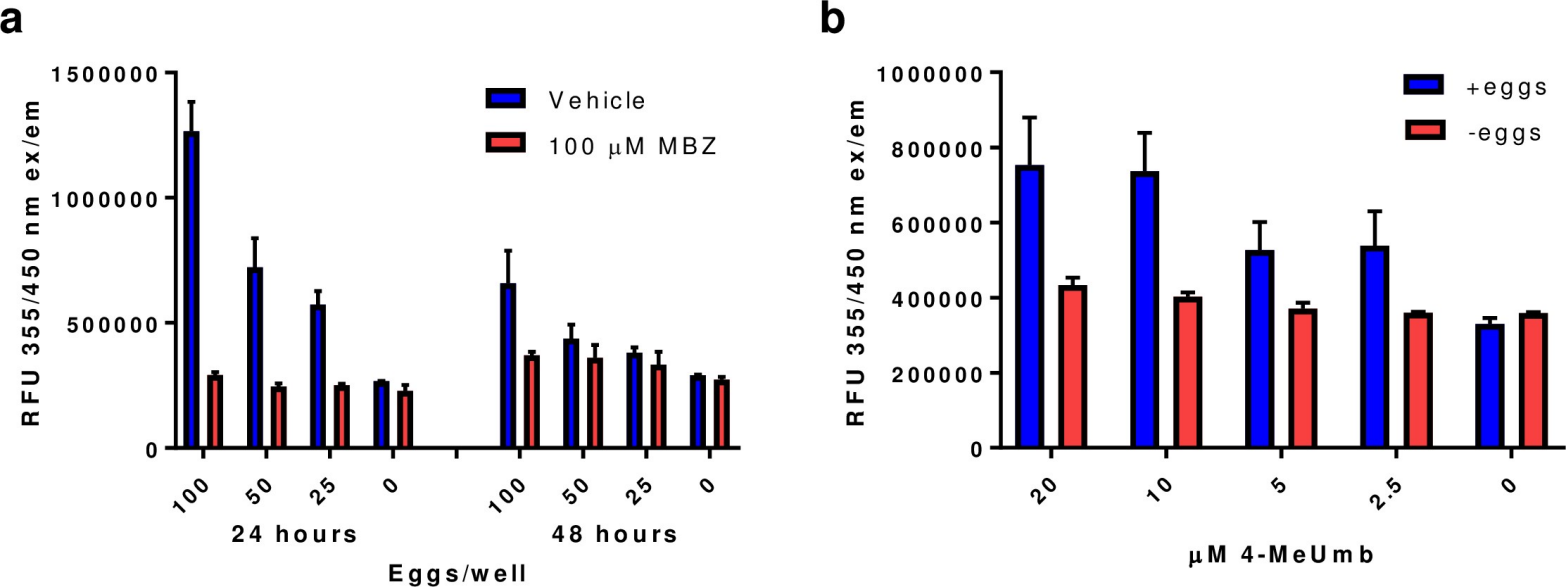
Assay optimization. **a.** Effect of *A*. *ceylanicum* egg number and mebendazole treatment time on the chitinase egg hatching assay. 100, 50, 25 and 0 eggs/well (20 μL) were treated with 1 μL of either 2 mM MBZ or DMSO vehicle for 24 or 48 hrs at 27°C followed by chitinase detection with 10 μM 4-MeUmb. **b.** Effect of 4-MeUmb concentration on the fluorescent signal produced by egg hatching. 20 μL of 50 eggs/well or water was incubated for 24 hrs at 27°C followed by chitinase detection with 20, 10, 5 or 2.5 μM 4-MeUmb.

In order to determine the effect of egg concentration on fluorescence signal, different concentrations of *A*. *ceylanicum* eggs were plated into 384-well plates with 4 replicate wells per treatment condition. The eggs were treated with either 100 μM mebendazole (MBZ) or 0.0125% DMSO vehicle and incubated for 24 or 48 h at 27°C. After incubation, the assay plates were examined by light microscopy. The majority of eggs (>90%) in the vehicle-treated wells had hatched whereas the MBZ-treated wells showed no egg hatching. 4-MeUmb was added to the assay plates at either 24 or 48 h and the fluorescence measured. At 24 h, there was a linear increase in fluorescent signal with increasing egg number in the vehicle-treated wells (r = 0.96). MBZ-treated wells showed background fluorescence (Fig 1A). The signal to background ratio ranged from 4.3-fold at 100 eggs/well to 2.3-fold at 25 eggs/well. At 48 h, the fluorescence signal was lower, *e*.*g*. at 100 eggs/well the signal was 50% lower relative to the same wells at 24 h. We next optimized the 4-MeUmb concentration. Either no eggs or 100 eggs/well were plated and incubated for 24 h at 27°C. After the incubation period, different concentrations of 4-MeUmb were added to the wells. The fluorescent signal was highest at 10 and 20 μM 4-MeUmb (Fig 1B). Accordingly, 10 μM 4-MeUmb was chosen for screening. An egg plating density of 75 eggs/well and 24 h of compound treatment were selected as optimal parameters for library screening. Examination by light microscopy of several wells after plating using the Multidrop Combi showed 45–63 eggs/well. After 24 hours incubation there were 43–55 larvae per well with 2–3 unhatched eggs/well.

### HTS

**Fig 2:**
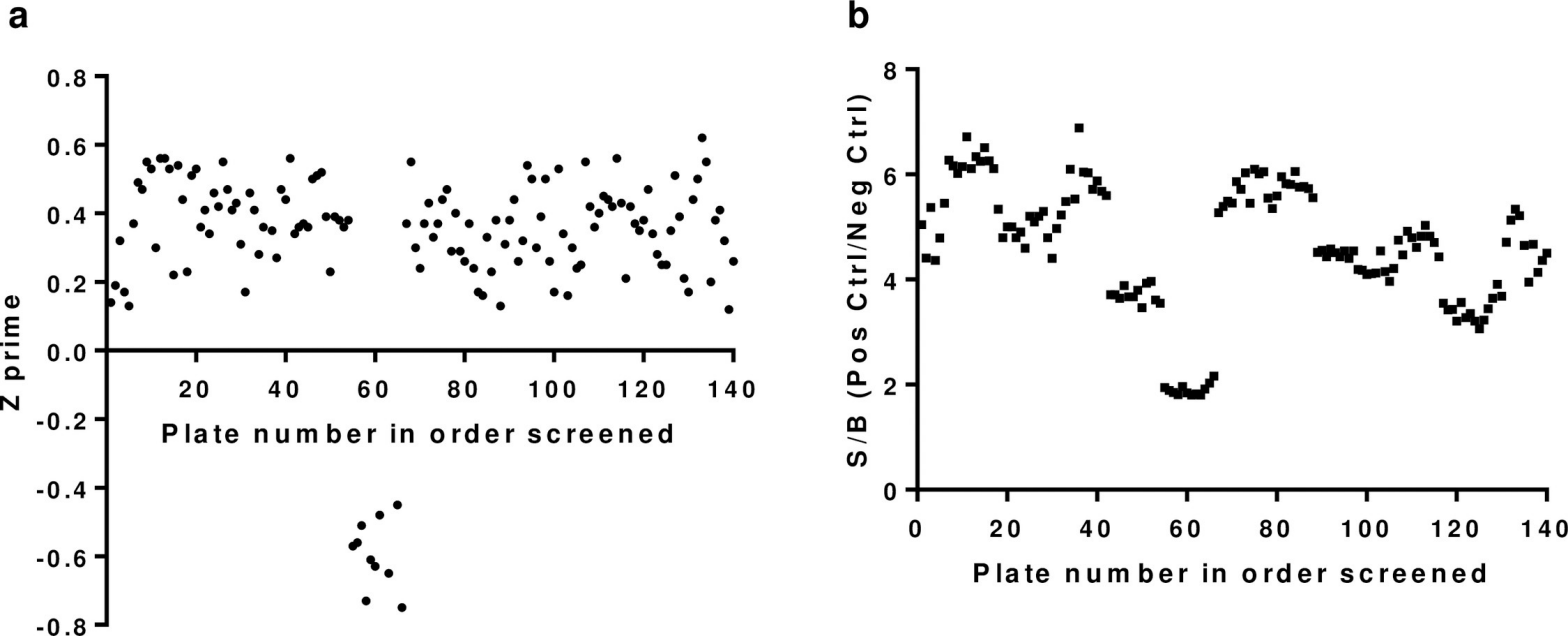
Primary screen quality. **a.** Z-prime value per assay plate screened. **b.** Signal/Background per assay plate screened.

Compounds (39,568) from several libraries were tested in concurrent screening runs in singlicate. Control wells of eggs in all screening runs (except one, attributable to poor egg hatching due to temperature fluctuations that occurred during transport of the isolated eggs to the screening lab) displayed acceptable Z’-factor values [15], ranging from 0.12 to 0.56, indicating an assay with good separation of negative and positive control wells (Fig 2A). Also, the signal/background (S/B) ratio ranged from 3.1 to 6.7 (Fig 2B) for all but one run.

**Fig 3:**
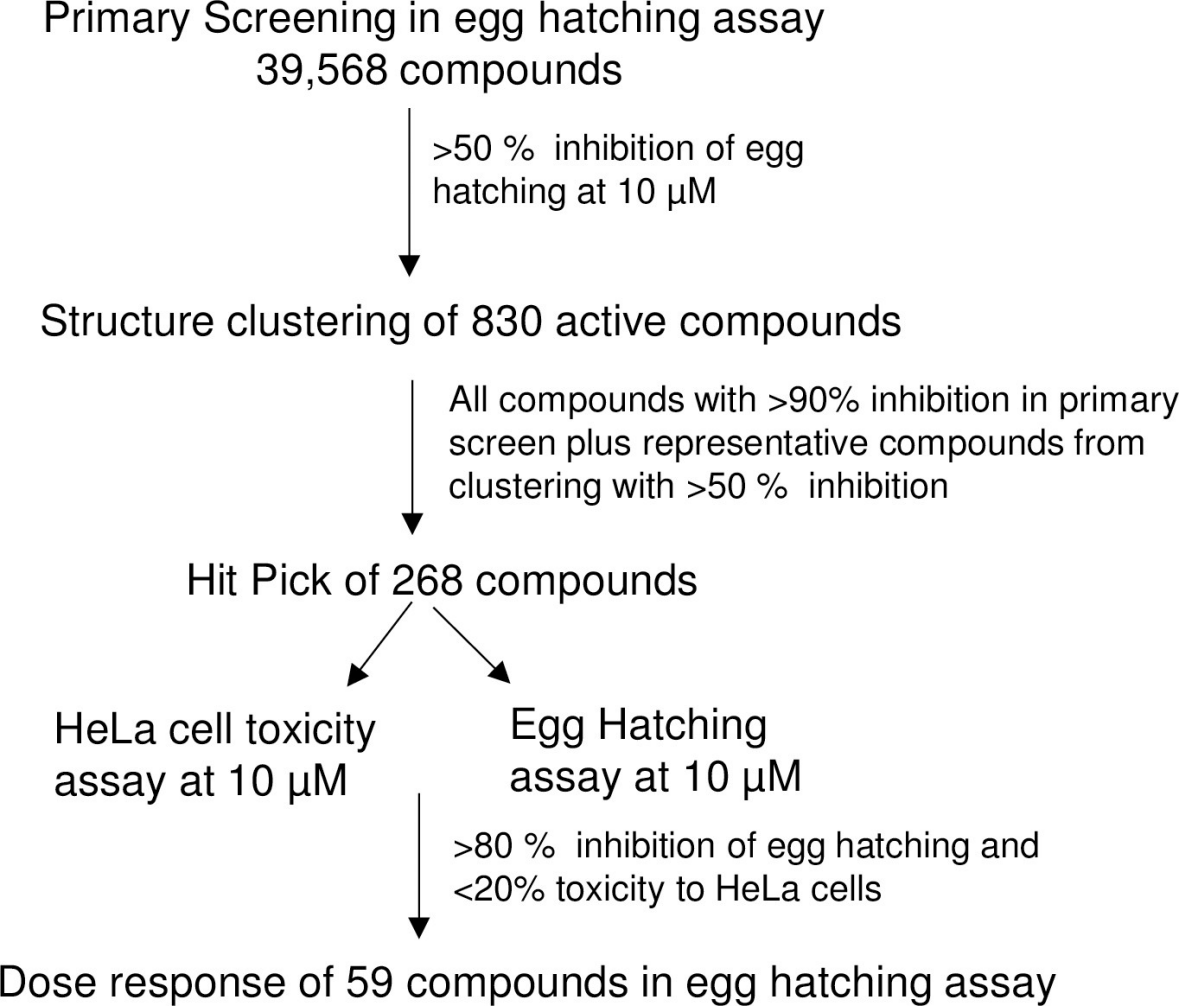
Cluster structures are followed by the number of members in parentheses. R groups are those positions most frequently substituted but all open valences or hydrogens sites may also be substituted.

From the primary screening of 39,568 compounds, we identified 830 bioactive compounds that inhibited egg hatching of *A*. *ceylanicum* eggs >50%. Of these, 132 compounds inhibited hookworm egg hatching by >90%. Among the libraries screened was a collection of extracts produced by endophytic microorganisms associated with tropical rainforest plants. Of these extracts, 10 inhibited egg hatching >70%. Visual inspection by substructure of the 830 active small molecule compounds showed in excess of 16 clusters (Fig 3) whereas the remaining compounds were classified as unrelated or singletons. Among the compounds identified were the benzimidazole anthelmintics albendazole, mebendazole, thiabendazole and fenbendazole. These clustered with another nine structurally similar benzimidazoles. A fifth known anthelmintic, levamisole, was also identified. The identification of these known anthelmintics among the libraries screened provides a useful internal validation of the HTS. The anthelmintic ivermectin was not active in this assay perhaps due to its inability to penetrate the egg shell.

**Fig 4:**
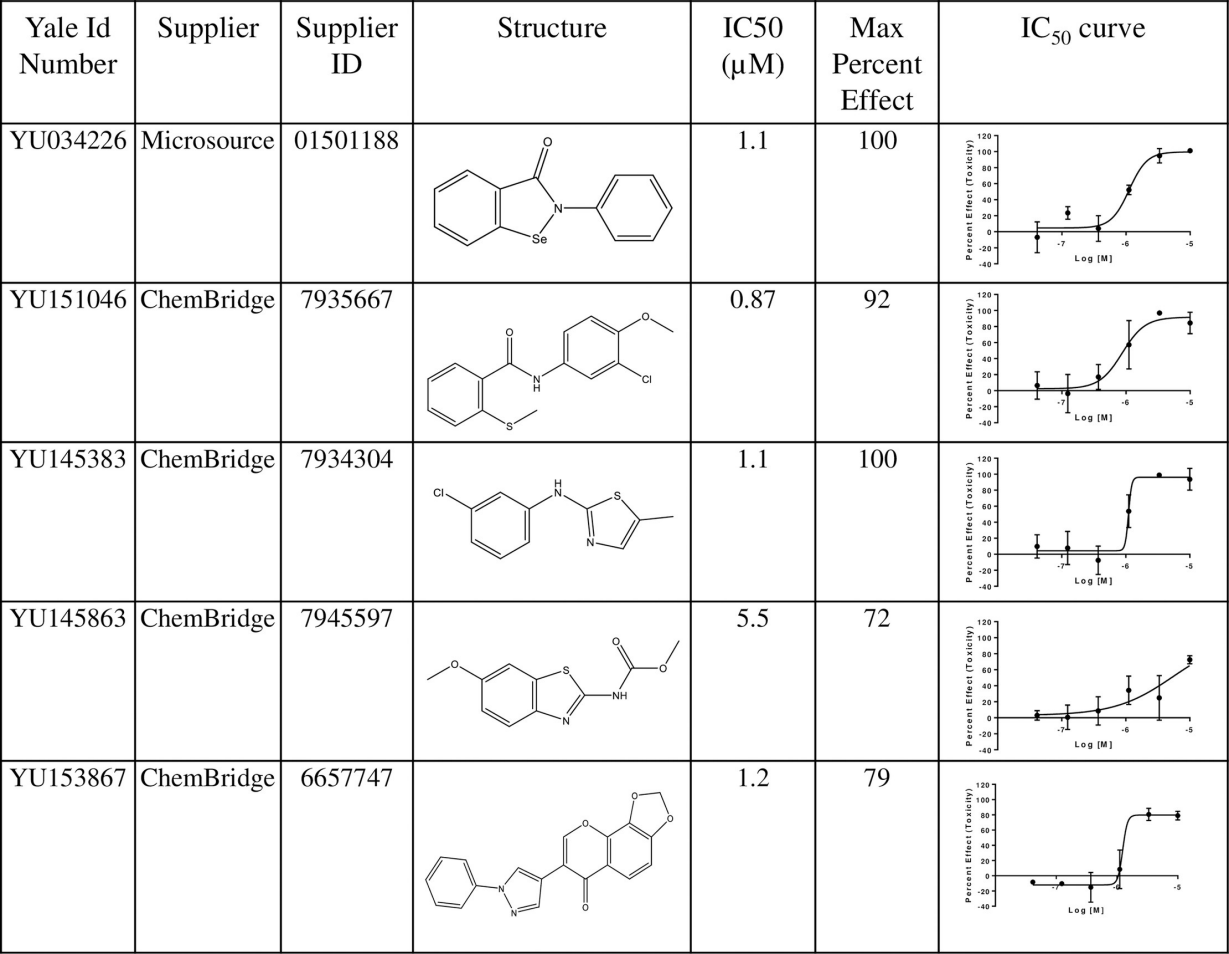
Schematic of the high throughput screening workflow.

Repeat testing was performed on 268 compounds in singlicate. These included all compounds with activity >90% and selected compounds from the identified structural templates that showed at least 50% effect in the primary screening assay. In addition, testing for toxicity against the HeLa cells was performed to rule out compounds that have the potential to be toxic to humans (https://doi.org/10.6084/m9.figshare.7961051.v1). Of the 268 compounds, 59 inhibited egg hatching >80% effect in both the primary screen and the repeat test and were not toxic to HeLa cells (<20% effect). Active compounds were visually confirmed by microscopic evaluation and none were acting as chitinase enzyme inhibitors. These compounds consisted of 11 known bioactive compounds (including known anthelmintics) and 48 synthetic compounds. The screen work flow is shown in Fig 4.

**Fig 5:**
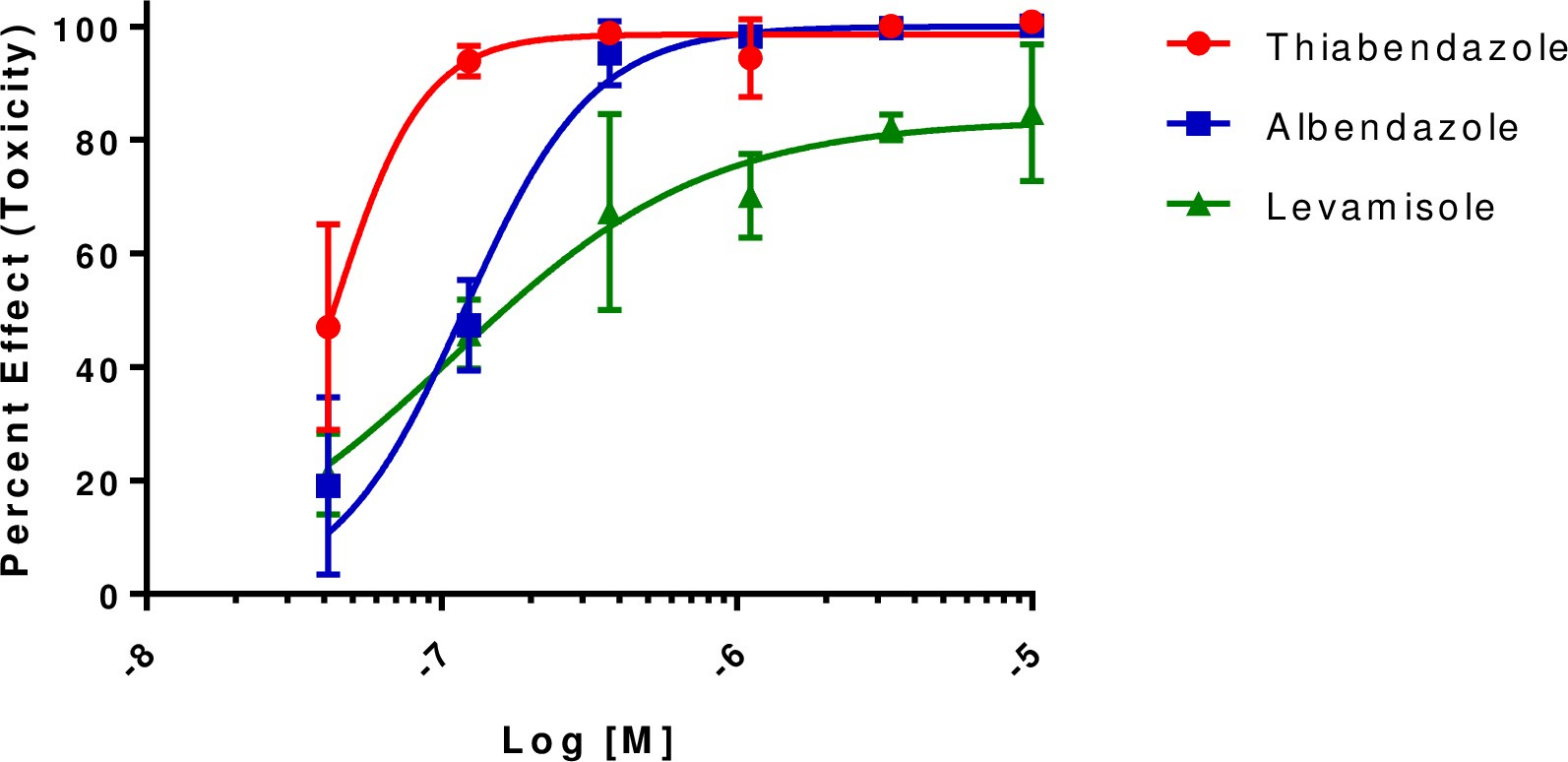
Half-maximal inhibitory concentration (IC_50_) values of known anthelmintics in the chitinase egg hatching assay. The IC_50_ values for Thiabendazole, Albendazole and Levamisole were 43 nM, 119 nM and 109 nM, respectively. Data from replicate experiments performed on two different days is plotted.

**Fig 6:**
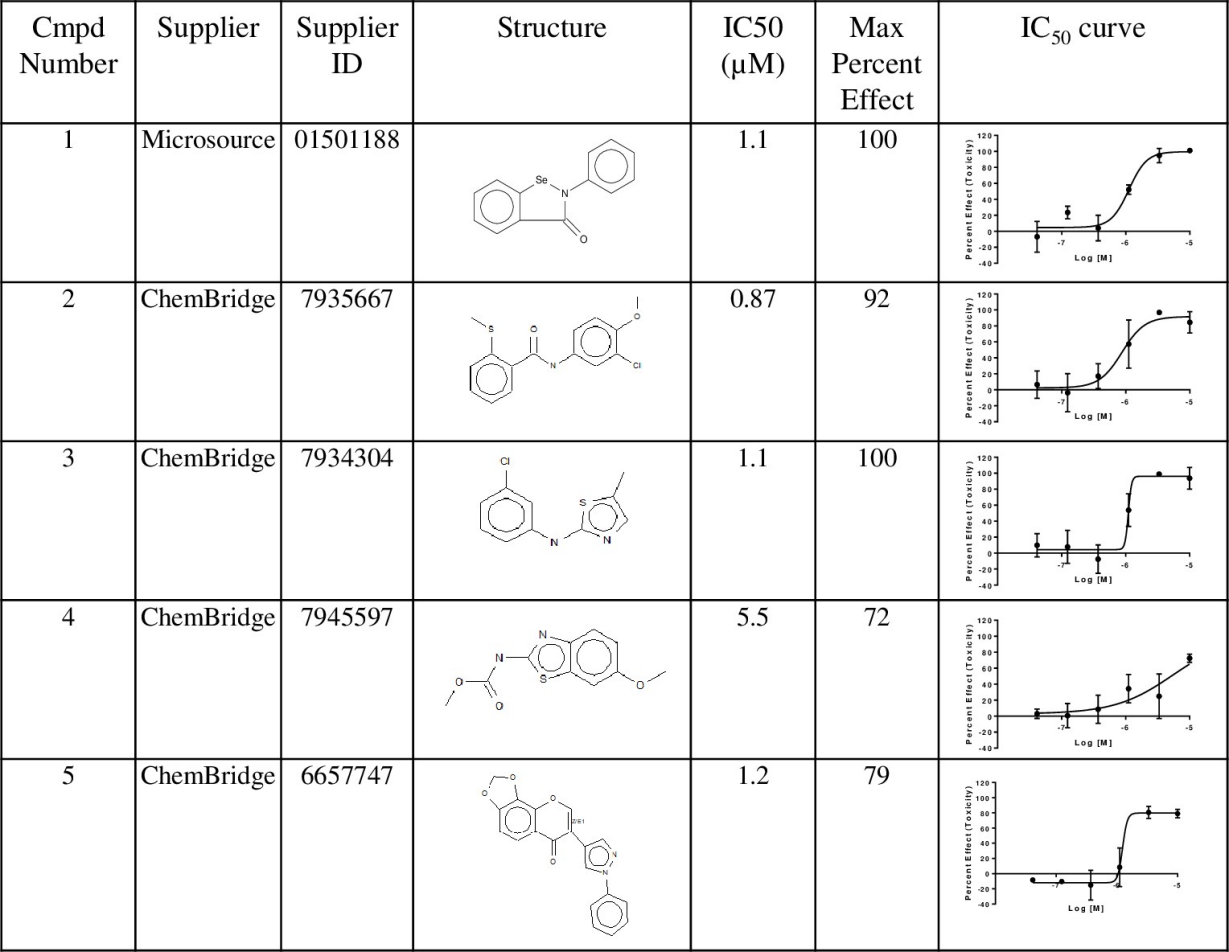
Top 5 most active non-benzimidazole related compounds identified by HTS screening.

IC_50_ values were determined for the 59 hit compounds in 2 different runs and ranged from 50 nM -9 μM with two compounds not showing a dose response due to low activity in both runs (https://doi.org/10.6084/m9.figshare.7971341.v1). As expected, known anthelmintics had potent IC_50_ values (Fig 5). The most active non-benzimidazole related compounds are shown in Fig 6. IC_50_ values ranged from 90 nM –6 μM.

## Discussion

Drug discovery strategies are based on biochemical target or biological function [16]. Discovery of anthelmintics mostly has been based on biological function [17]. Our screening strategy was based on inhibiting the biological function of egg hatching to L1 stage larvae. Compounds that are impermeable to the egg or have mechanisms of action that do not impact egg hatching will be inactive in this assay. In addition to directly affecting the desired biological function, any compounds discovered as active are permeable to the organism unlike active compounds discovered in a biochemical target screen which may or may not be permeable to the organism.

**Fig 7:**
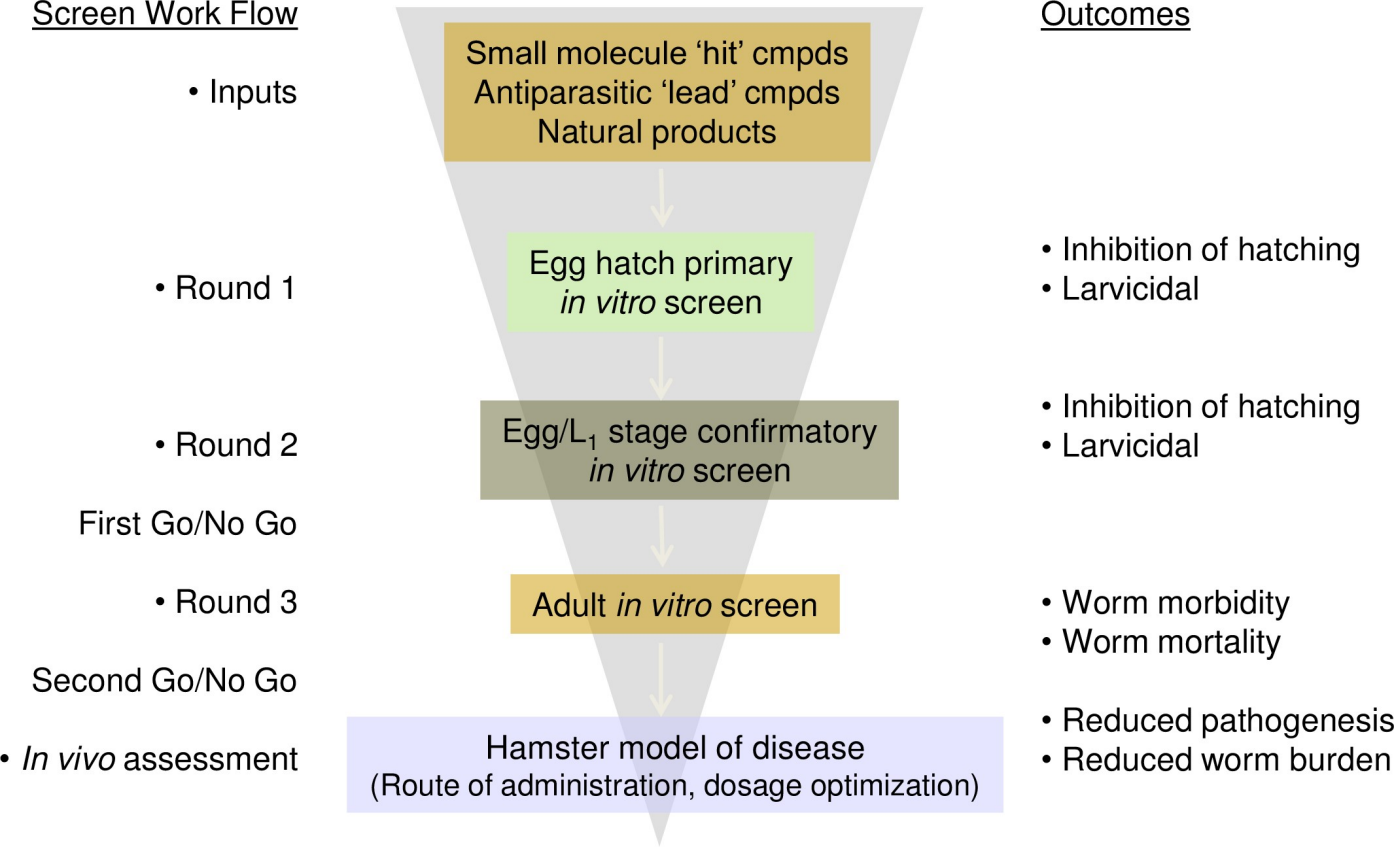
Screen Work Flow.

Whole organism HTS screens of helminths have been reported in the literature. Some screens scored activity of compounds visually using a microscope [18–20]. Others used an image-based approach [21]. In one case, a fluorescent reporter assay was used [22]. We developed a plate reader based screening approach using a fluorogenic chitinase substrate to detect *A*. *ceylanicum* hookworm egg hatching. The use of a chitinase substrate to detect egg hatching has been used successfully in several nematode HTS screens against *C*. *elegans* [23, 24] and may be applicable to other helminth parasites in which chitinase is released upon egg hatching as well as *Plasmodium* spp. which employ chitinase to penetrate the midgut of their mosquito vectors [25]. High-throughput assays such as this allow the rapid evaluation of small molecule compounds for anthelmintic activity and prioritize compounds for further *in vitro* and *in vivo* testing. From our HTS screen of 39,568 compounds, we have prioritized for further investigation 5 non-benzimidazole related compounds displaying low micromolar toxicity to *A*. *ceylanicum* eggs with little toxicity to mammalian cells. These 5 compounds have not been extensively studied previously. Compound 1 (ebselen) has not been included in any other bioassays. Compounds 2, 4, and 5, were in 1, 1, and 12 bioassays, respectively, and were found to be inactive in all. Compound 3 was examined in one bioassay and found to be active (Bioassay High AID1949: Throughput Screen of 100,000 compound library to Identify Inhibitors of *Mycobacterium tuberculosis* H37Rv). Future experiments will be designed to 1) systematically assess the nematicidal activity of these compounds against adult stage *A*. *ceylanicum* hookworms in culture and 2) define their chemotherapeutic potential *in vivo* by treating hookworm-infected hamsters (Fig 7). Information gained from these experiments may directly contribute to the development of new drugs for the treatment of human hookworm disease.
